# The Impact of Change Management in the Greek National Health System: A Quantitative Study

**DOI:** 10.7759/cureus.45574

**Published:** 2023-09-19

**Authors:** Alexandra Vlassi, Georgios Tzikos, Theodoros Dardavesis, Olga Zioga, Konstantinos Papadimitriou, Theodosios Papavramidis

**Affiliations:** 1 Laboratory of Hygiene, Social & Preventive Medicine and Medical Statistics, School of Medicine, Faculty of Health Sciences, Aristotle University of Thessaloniki, Thessaloniki, GRC; 2 1st Propaedeutic Department of Surgery, AHEPA University Hospital, Thessaloniki, GRC; 3 Department of Surgery, School of Medicine, Faculty of Health Sciences, Aristotle University of Thessaloniki, Thessaloniki, GRC

**Keywords:** greece, healthcare system, qualitive study, economic crisis, change management

## Abstract

Introduction: Serious changes took place in Greece due to the economic crisis of 2008, which led to significant changes in Greece’s health sector. The reforms and changes that were made to the National Health System (NHS) aimed to improve it, provide quality services to its users, and adapt it to Greece’s new external environment. The aim of this study was to assess the management of these changes by the leadership of the NHS and to examine their effectiveness.

Material and methods: The survey was based on quantitative research methods, using a questionnaire as a research tool. The sample population comprised employees of a tertiary-care public hospital in northern Greece.

Results: We recruited a sample size of 100 participants for the survey. The questionnaire’s Cronbach’s alpha was equal to 0.724. The overall change management was moderate, and Greece’s NHS did not follow a specific change-management model that provided principles facilitating the success of the effort. The most serious problems identified by using the questionnaire were the following: the changes were not structured and clear, the management set unrealistic goals and objectives, the changes to the NHS were made without the appropriate financial support, and there is uncertainty about the future of health care in Greece.

Conclusion: The present study showed that changes cannot be made randomly; rather, they require careful planning and organization. Further changes, continuous feedback, and development are required to improve the status of Greece’s healthcare system.

## Introduction

Public organizations such as the public health sector should be continuously sustainable [1], as they are critical for the socio-economic development of a country. To achieve this sustainability, the sector should advance the consequent necessary changes in its management model. Change management is a complex and demanding process, and it is considered one of the primary concerns in healthcare management [2]. In general, management in the public health sector follows the same principles as those of change management in other types of organizations. The main difference concerns hierarchical structures, which are more distinct in the public health sector than in other organizations [3]. Distributive leadership is particularly effective in the change process in the health sector [4]. Furthermore, to maintain the smooth functioning of the public healthcare organization, managers and employees must be properly prepared to acquire the necessary knowledge, skills, and training regarding the changes they are going to experience [5]. In addition, leaders of an organization must understand the process of change and its related issues to ensure a successful outcome. Awareness programs and clear communication between public officials and the organization can also aid in successful change management. A healthcare organization must utilize the strengths of change management, which aims to incrementally improve existing organizational capabilities, empower changing conditions, and continuously support change-management leaders [6].

Greece’s healthcare system has faced serious problems for many decades in terms of organization, funding, and quality of services. These persistent and unresolved problems have not only led to financial and operational difficulties but also citizens’ low satisfaction with the health sector. The economic recession of 2008 brought a series of structural reforms in hospital care such as budget restrictions in public hospitals, restructuring of health units, computerization of financial services, and conducting of central procurement. According to the memorandum signed with the European Union and the International Monetary Fund, Greece agreed to implement a series of policy measures aimed at reducing existing weaknesses in the healthcare sector, enhancing the efficiency of hospitals, and improving health services [7].

The success of the above-mentioned reform effort depends partly on the successful implementation of policies to improve the utilization of available resources, and the smooth implementation and acceptance of these changes by all healthcare professionals. Hospital managers play a central role in achieving this goal. Moreover, public hospital reforms require close cooperation and coordination between planning and implementation policies, as well as matching the scale and pace of reforms with their implementation capacity. Over time, through iterative evaluation, review, and adaptation, the Ministry of Health can identify how efficiency could be managed in the sector and move towards more consistent policies, implementation of standards regarding service delivery, and fair and equal access to the entire Greek hospital system to all citizens [8].

The purpose of this study was to evaluate the change management that has been applied during the introduction of changes to the Greek healthcare system, to examine whether a specific change-management model has been applied, and to assess whether there has been proper planning and preparation before the introduction of changes.

## Materials and methods

Type of research and research tool

The survey was of a quantitative type, using a questionnaire as a research tool. The questions were based on change management theory and, more specifically, on the Canadian Health Services Research Foundation (CHSRF) model. The questionnaire consisted of two parts. In the first part, the participants were asked four questions related to their demographics, including age, gender, education, and professional position. In the second part, they were asked to answer 23 questions related to change management. Answers were given according to a five-point Likert scale (5=strongly agree, 4=agree, 3=neither agree nor disagree, 2=disagree, 1=strongly disagree). The questionnaire was introduced by the researchers after consensus about the questions that should be included in order to address all aspects of the changes in the Greek NHS. Because the questionnaire was newly designed and provided for the first time, a pilot study including 15 participants was conducted aiming to evaluate the reliability of the questionnaire and reveal any difficulties concerning the conceptual equivalence and the semantic concept that have to do with the level and complexity of the language used in the questionnaire, so that it is understandable by the majority of the target population.

Sample and sampling

The study sample consisted of 100 employees randomly selected from a tertiary-care public hospital, AHEPA University Hospital of Thessaloniki, in the Prefecture of Thessaloniki, North Macedonia, Greece. The first author chose convenience sampling, as he works at that same public hospital. There was no restriction on the demographic characteristics of the target population. The only inclusion criterion to take part in the survey was that participants were healthcare professionals or administrative staff working in the Ministry of Health. All participants signed an informed consent form before participating in the study. The sample size was calculated after considering the total number of eligible employees in the hospital (768) and setting the confidence level at 95% and the margin of error at 10%. This gives back a sample size of 86 participants, but since several authors/researchers regarded 100 participants as the minimum sample size when the population is large, we chose to include 100 participants. This reduced our margin of error to 9%.

Study outcomes

The primary outcome of this study was to evaluate the changes introduced to Greece’s National Health System (NHS) and to clarify whether there were any problems regarding the implementation of this change management.

CHSRF model

The CHSRF model relies on information-based change management and aims to develop leadership to support change [9]. Its main advantage is that it describes the change in a concrete way. It is a practical model for managing change that contains four stages: planning, implementing, propagating, and sustaining change. At all stages of change planning, change initiators should seek to understand the context and dynamics of change and to determine change readiness and capability. To successfully implement change, change initiators must understand the stage of change and take the necessary steps to manage the change [10].

Statistical analysis

All collected data were saved in a Microsoft Excel spreadsheet (Microsoft Corporation, Redmond, Washington, United States). Statistical analysis was conducted using IBM SPSS Statistics for Windows, Version 21.0 (Released 2012; IBM Corp., Armonk, New York, United States) after extracting the data. The normality of data distribution was assessed using the Kolmogorov-Smirnov test. Participants were described according to their sociodemographic characteristics (e.g., sex and age). The results of continuous variables were presented as means ± standard deviations (SDs) when normality was assumed, or as medians with their respective interquartile range (IQR) (Q25-Q75) when the data were skewed. Categorical variables were presented as counts and percentages. As Cronbach’s alpha was calculated to be to 0.724, the research results were considered reliable. In addition, statistical significance was set at p<0.05. Finally, we decided to present the results of the Likert scale included in our questionnaire as means ± SDs and not as medians and IQRs, because these parameters are considered easier to be understood by the readers. In addition, we calculated the average mean (adding all the means and dividing the result by the number of the means (23 means)) to define the cut-off point for a disagreement decision.

## Results

We recruited a sample size of 100 people for the survey, with their demographics listed in Table 1.

**Table 1:** Participants’ demographics

Demographic parameter		N=100
Gender	Male	17
	Female	83
Age (years)	<30	12
	31-40	37
	41-50	46
	51-60	4
	>61	1
Education	High school	6
	Technological degree	40
	University degree	36
	Master	14
	PhD	4
Occupation	Doctors	14
	Nursing staff	59
	Administrative staff	27

Most of the participants were women (83%), 41-50 years of age (46%), held a technological education degree (40%), and were employed as nursing staff (59%). Table 2 shows the results of the change-management questionnaire filled out by the participants.

**Table 2:** Results of the questionnaire related to the management change. SD: Standard Deviation Answers were given according to a 5-point Likert scale (5=strongly agree, 4=agree, 3= neither agree nor disagree, 2=disagree, 1=strongly disagree)

Type of change management	Mean	SD
The Administration in the National Health System does not have a precise organizational vision for the future of the changes that have been made.	2.98	0.82
Members of the management do not agree with the strategy being implemented.	3.19	0.83
Changes were made after a detailed analysis of the real situation prevailing in the National Health System.	2.78	0.93
The changes that were made were structured and clear	2.06	0.91
Management clearly defined the goal of each change.	3.07	0.81
Employees had available all the details of every change made to the National Health System	2.73	0.84
When management faced obstacles in implementing the changes, it abandoned its original vision.	2.22	0.89
Management has set unrealistic goals and objectives, so many changes have been left in the middle and not completed.	3.94	0.75
During the implementation of the changes, management did not consider the potential risks and did not have an alternative action plan.	2.96	0.83
Management did not take into account the resistance that employees in the organization may have shown due to changes	3.45	0.91
Management members who implemented the changes lacked proper training and knowledge on change management	2.29	0.95
The members of the Management who implemented the changes did not have proper training and knowledge on how to deal with the resistance to changes that health workers would project.	3.75	0.86
Management is constrained by its personal interests and perpetuates an authoritarian system based on hierarchy.	4.10	0.87
Management is not reliable and its opinion has no weight to the health workers.	2.97	0.73
Management did not clarify the objectives of the changes to employees	3.56	0.88
During changes, management provided official channels of communication with employees about upcoming changes.	2.96	0.99
During changes, management made the employees involved and strengthened their degree of involvement in the project as they were the ultimate responsible for its implementation.	2.19	1.11
Employees who are expected to implement changes are not involved in making and preparing decisions.	4.12	0.99
Contradictory and disrupted communication from leadership to employees leads to repeated misunderstandings, making leadership untrustworthy to employees.	3.16	0.84
After the changes are implemented, the duties of the employees are not clear, in a formal way	2.25	0.92
Today there is uncertainty about the future situation in the field of Health. It is not clear who will do a task and in what way.	4.22	0.92
Changes in the National Health System were made without proper financial support.	4.48	0.94
There are no plans and ready-made procedures for the changes in the field of Health and so there is no systematic implementation of the projects.	2.59	0.76

The overall change management was calculated to be 2.93 (SD=0.91). This expresses the overall opinion of all the responders regarding the way the management change was performed. Meanwhile, the average mean was 3.13. The fact that the overall mean (2.93) is quite close to the average mean (3.13) of this five-point Likert scale, means that, overall, the participants neither agreed nor disagreed with the way changes were applied. The most serious problems identified by the survey, by the use of the 23 questions, and evaluating the deviation of the score for the average mean value of 3.13 were those with a deviation towards 1 (negative trend) and those with a deviation toward 5 (positive trend). In the first category (negative trend), we identified that (i) the changes were not structured and clear (mean=2.06, SD=0.91) and (ii) during changes, management failed to make the employees involved or strengthen their degree of involvement in the project (mean=2.19, SD=1.11). In the second category (positive trend), we identified that (i) the management set unrealistic goals and objectives, and many changes had not been completed (mean=3.94, SD=0.75), (ii) the management was limited by its personal interests and perpetuated an authoritarian system based on hierarchy (mean=4.10, SD=0.87), (iii) the employees who are expected to implement changes did not participate in decision-making and preparation (mean=4.12, SD=0.99), (iv) the changes to the NHS were made without the appropriate financial support (mean=4.48, SD=0.94), and (v) there is uncertainty today about the future of healthcare (mean=4.22, SD=0.92). The above are presented in Table 2.

## Discussion

In this study, we have evaluated for the first time the impact of the change management applied in the Greek NHS as perceived by its employees. The overall impact could be considered neutral (mean: 2.93 (SD:0.91), neither agree nor disagree), revealing that probably more actions and time are needed for these changes to have a favorable effect on the Greek NHS. More precisely, one of the most serious problems identified was the fact that the management set unrealistic goals and objectives, and many changes had not been completed, indicating that the conditions for the implementation of reforms are still immature. However, the members of management who implemented the changes were found to have the proper training and knowledge on change management.

A further worrying finding was the fact that the range of questions (Management is constrained by its interests and perpetuates an authoritarian system based on hierarchy, mean: 4.10 (SD:0.87), Employees who are expected to implement changes are not involved in making and preparing decisions, mean: 4.12 (SD:0.99)) highlighted that management staff was trying to implement changes in their own self-interest, while at the same time, key staff was being sidelined away from the center of decision-making. Therefore, the uncertainty for the future of healthcare reported by the participants could be considered both reasonable and expected (mean: 4.22, SD: 0.92).

The international literature on change management reflects its two basic functions: planned change management and emerging change management. Planned change management dominates the academic literature, largely because of the work of Kurt Lewin [11]. Throughout the process of planned change management, a series of pre-planned steps are used [12]. The planned change approach recognizes that in order to successfully adopt new behaviors within an organization, old behaviors must be abandoned. Unlike planned change, emerging change emphasizes a “bottom-up” approach to managing change. While the planned change model highlights the pre-planned processes and objectives that underscore the role of management, the emerging change approach argues that the pace and nature of change are so fast and complex, respectively, that senior management can encounter difficulties in identifying changes and devising strategies to address them in a timely manner. As a result, managers must cede part of their decision-making power to employees and act as facilitators of change, as opposed to controllers of change [13]. Although these models are applied in a variety of settings, their use in the literature does not prove their universal applicability [14].

In some cases, the best strategy for organizations to adequately manage a change falls somewhere in between these two theories, which requires intelligent integration based on the specific circumstances of the organization. To achieve successful change, an interaction of factors is required, including organizational (i.e., internal) and environmental (i.e., external) conditions leading to change [15]. This interaction of factors is vital for the Greek NHS, where serious problems were identified by the survey, such as not structured and unclear changes, unrealistic goals and objectives set by management, uncompleted change processes, lack of proper training and knowledge of managers regarding change management, limitations in management and improper leadership style (an authoritarian system based on hierarchy), absence of employees’ involvement in decision-making and preparation of change, lack of financial support, and uncertainty about the future of healthcare. All these problems should be solved through the implementation of both emerging and planned change management.

As models of emerging change management are mostly used in structural changes at a higher level, they can be applied to changes in Greece’s broader NHS rather than to micro-level changes in healthcare facilities. Although these models are applied in a variety of settings, their use in the literature does not prove their universal applicability [15].

Lewin constructed four theories that together lead to understanding and establishing a framework of planned change: field theory, group dynamics, action research, and the three-step model (Unfreeze-Change-Refreeze). Although these theories are often considered independently as separate themes of Lewin’s work, they form a unified whole where each element facilitates the understanding of planned change [16].

In addition to planned and emergent approaches to change management, which are derived primarily from the business literature, several other models of change management have recently been developed in the healthcare context. The Organizational Model for Transformative Change in Health Systems developed by Lukas et al. defines healthcare organizations according to four elements: mission, vision, and strategies; culture; business functions and processes; and infrastructure [17]. A change in any of these four components constitutes a change in the organization or healthcare system according to this model. To facilitate the change process, the model suggests five key elements of transformational change in healthcare organizations: motivation for transformation, leadership commitment to quality, improvement initiatives that help actively engage staff in meaningful problem-solving, alignment to achieve organizational coherence at the level of goals by allocating resources and actions at all levels of the organization, and integration and bridging of traditional intra-organizational boundaries between individual components.

Lukas et al. present these five interrelated elements as necessary to successfully implement change in healthcare organizations [17]. To achieve successful change, leaders must recognize the need for change and help staff actively participate in supporting short-term improvement initiatives to perpetuate their results. In addition, leaders must take steps to ensure initiatives are aligned with broader organizational goals.

According to the review of the literature, the process of change involves three stages: planning, implementing, and maintaining change. The first stage in the process of managing change (planning) involves facilitating the mental preparation of all stakeholders necessary to achieve a successful change. Planning also involves developing a common framework and understanding of the current situation. Although, theoretically, this can be done effectively by using a map that depicts the process, the present study showed that changes made in the health sector were not accompanied by proper preparation.

Another critical component of preparing for change in Greece’s health system is planning cultural change. Today, Greece’s health system uses, in most cases, a highly restrictive and traditional approach to health care [18], rather than placing the patient at the center of the service delivery model [19]. Changing this policy requires redesigning healthcare settings, and involving patients and healthcare providers. In Greece, healthcare policymaking must focus on improving the effectiveness and efficiency of the country’s health system. Policies often conflict with existing practices and traditions, and make a change design difficult. In particular, the results of the present survey showed that when reforming policies are made, management should be aware of their potential outcomes and communicate the vision for the change [20]. A key takeaway from both theory and research is that strong leadership is fundamental to successful change. Effective leaders must maximize every opportunity and work with all stakeholders to legitimize the necessary changes and encourage all parties to offer their support.

The next phase of the process of change is the implementation phase, which involves carrying out the processes and activities necessary to make the change happen. A key challenge for both policymakers and healthcare professionals is to create a culture in which the top priority of healthcare professionals is to provide patients with care of the highest quality [21].

The last stage of the change process is to maintain the change. The sustainability of the change depends on the endurance of the new methods and performance levels in the organizational environment. It is not only the processes that change but also people’s thinking and attitudes, which will have to change permanently to create a new culture. The transformation should be able to withstand challenges and possible reactions, and be open to further improvements in the future. This resilience is particularly important because the benefits gained from new practices diminish over time and thus reduce the sustainability of the change.

The present study showed that the participants had the opinion that the management was not effective and many serious problems were identified through the change-management process, according to their views. To achieve an effective change-management process, managers must be able to offer support to all healthcare professionals, communicate the vision behind the change, understand the culture of the organization, and carefully align the desired changes with the existing perception of needs within the organization. 

This study has some limitations. First, the relatively small sample size was recruited from only one tertiary-care public hospital. However, this was considered adequate based on the total number of the target population. Moreover, the selection criteria were loose, and therefore heterogeneity between the participants may be present. However, the major strength of this study is the fact that it is the first attempt to evaluate the change management that had been applied during the introduction of changes to the Greek NHS from the point of view of those who work in it. At this point, it is essential to highlight that in ordinal scales, such as the Likert scale that was used in our questionnaire, the differences between the responses “strongly agree”, "agree”, “neither agree nor disagree”, “disagree”, “strongly disagree” may not be necessarily equal and it should not be assumed that they are equidistant, although the numbers assigned to those responses are [22]. Therefore, caution should be given to the interpretation of these results. Finally, this study is the first step in an ongoing process of evaluating the changes that have taken place in the Greek NHS to identify the first reactions on the part of its employees, which will then form the basis for the conduct of studies aiming at identifying the feedback on how to improve the quality care in the healthcare system.

## Conclusions

The present study showed that changes cannot be made randomly, as they require careful planning and organization. Leadership plays an important role in ensuring that initiatives for change are aligned with broader strategic goals. As research results showed, stakeholder involvement is a matter of great importance for managing change throughout the whole change process. Communication, workflow analysis, and integration as well as chances for education and training also play important roles in managing change. The results of the survey will help to evaluate the management of the changes introduced to Greece’s NHS. These important structural reforms should be monitored with updated data during the coming years to assess their effectiveness and impact on the operation and performance of Greek hospitals.
